# Prenatal Vitamin D Levels Influence Growth and Body Composition until 11 Years in Boys

**DOI:** 10.3390/nu15092033

**Published:** 2023-04-23

**Authors:** Julia Sanguesa, Sandra Marquez, Mariona Bustamante, Jordi Sunyer, Carmen Iniguez, Jesus Vioque, Loreto Santa-Marina Rodriguez, Alba Jimeno-Romero, Matias Torrent, Maribel Casas, Martine Vrijheid

**Affiliations:** 1 ISGlobal, 08036 Barcelona, Spain; 2 Spanish Consortium for Research on Epidemiology and Public Health (CIBERESP), 28029 Madrid, Spain; 3 Universitat Pompeu Fabra (UPF), 08002 Barcelona, Spain; 4 Department of Statistics and Operational Research, Universitat de València, 46010 València, Spain; 5 Alicante Institute for Health and Biomedical Research (ISABIAL-FISABIO Foundation), 03010 Alicante, Spain; 6 Nutritional Epidemiology Unit, University Miguel Hernandez, 03202 Elche, Spain; 7 Biodonostia Health Research Institute, Group of Environmental Epidemiology and Child Development, 20014 San Sebastian, Spain; 8 Ministry of Health of the Basque Government, SubDirectorate for Public Health and Addictions of Gipuzkoa, 20010 San Sebastian, Spain; 9 Preventive Medicine and Public Health Department, University of the Basque Country, 48940 Leioa, Spain; 10 IB-SALUT, Area de Salud de Menorca, Menorca, Spain

**Keywords:** epidemiology, pregnancy, paediatrics, vitamin D, growth, body mass index, fat mass, overweight

## Abstract

Background: Gestational vitamin D levels may influence offspring growth and modulate adipogenesis. Findings from prospective studies are inconsistent, and few have evaluated the persistence of these associations into late childhood. Objective: To examine the association between prenatal vitamin D levels and growth and adiposity in late childhood. Methods: We included 2027 mother–child pairs from the INMA birth cohort. 25-hydroxyvitamin D_3_ (vitamin D_3_) levels were measured in serum at 13 weeks of pregnancy. Sex- and age-specific body mass index z-scores were calculated at 7 and 11 years, overweight was defined as z-score ≥ 85th percentile, and body fat mass was measured at 11 years. Z-score body mass index (zBMI) trajectories from birth to 11 years were identified using latent class growth analysis. Results: The prevalence of vitamin D_3_ deficiency (<20 ng/mL) was 17.5%, and around 40% of the children had overweight at both ages. Associations between vitamin D levels and outcomes differed by sex. In boys, maternal vitamin D_3_ deficient status was associated with higher zBMI, higher fat mass percentage, higher odds of being overweight, and with an increased risk of belonging to lower birth size followed by accelerated BMI gain trajectory. In girls no associations were observed. Conclusion: Our results support a sex-specific programming effect of early pregnancy vitamin D_3_ levels on offspring body composition into late childhood observed in boys.

## 1. Introduction

Vitamin D deficiency during pregnancy has been internationally recognized as a substantial public health concern. This is mostly due to its high prevalence among the population and is especially worrying in pregnant women and newborns because of its association with poor health outcomes [1,2]. Vitamin D deficiency has been identified as a potentially modifiable early risk factor for the development of a range of conditions, including obesity [3]. Similarly, there is great concern about the high prevalence of being overweight in childhood, which often persist into adulthood, hence increasing the risk of suffering from related conditions [4]. Sedentarism, high energy intake and a poor-quality diet are known to be major contributing factors to this alarming trend, but many other factors have been identified [5].

Several studies have demonstrated the association between deficient vitamin D levels during pregnancy and adverse fetal outcomes such as preterm birth or low birth weight [6], which have been associated with an increased susceptibility to develop diseases later in life, including cardiometabolic disease [7]. In addition, several biologically plausible mechanisms support the association between vitamin D and infant growth and adiposity, starting with the well-known role of vitamin D on musculoskeletal health [8], but also including its role on lipolysis and adipogenesis [9,10], although the exact mechanisms are not well established yet. Furthermore, vitamin D has been postulated as a modulator of adipose tissue inflammation, a key element in metabolic disorders [11]. Polymorphisms in the vitamin D receptor (VDR) gene have been linked to adiposity phenotypes [12] and obesity [13].

Despite the evidence mentioned above and the findings from cross-sectional studies linking low vitamin D levels to overweight or obesity risk in children and adolescents [14,15,16], there is not enough evidence to support a causal role of prenatal vitamin D levels in the development of obesity in children. While some prospective studies found no association between vitamin D levels during pregnancy and the risk of being overweight or a higher fat mass in offspring [17,18,19,20], results from other studies do support this association [21,22,23]. In our population-based birth Spanish cohort, we previously reported that low (<20 ng/mL) vitamin D levels during pregnancy increased the risk of prenatal and early postnatal overweight, although the association was attenuated at 4 years of age [21]. A recent systematic review and meta-analysis of interventional studies showed that results from previous randomized control trials (RCT) are also inconsistent [24]. However, the meta-analysis revealed that vitamin D supplementation during pregnancy or early life was associated with lower BMI and zBMI from 3 to 6 years [24]. In addition, some studies have identified factors that may influence the association between prenatal vitamin D and offspring growth and body composition, including pre-pregnancy body mass index [23,25], maternal smoking status [26] and the sex of the child [19,22].

From this available evidence, it is clear that very few studies, whether observational cohorts or RCTs, have been able to follow children beyond early childhood [19,26]. No previous studies have studied BMI trajectories, which integrate multiple aspects of growth over a period of time, such as birth size, BMI gain velocity and BMI peaks, permitting a more accurate identification of children at higher disease risk than BMI assessed at a single time point [27].

Therefore, in the present study, we aimed to investigate whether maternal vitamin D levels in early-mid pregnancy are associated with BMI, BMI trajectories, overweight risk, and body fat mass up in children up to 11 years of age. We also aimed to evaluate the effect modification by previously reported factors (sex, maternal pre-pregnancy BMI, maternal smoking) and by the child’s genetic susceptibility for high BMI using polygenic risk scores, which, to our knowledge, no study has analyzed before.

## 2. Materials and Methods

### 2.1. Population of Study

For this study, we included data from four regions of the Spanish population-based birth cohort INMA (INfancia y Medio Ambiente, Environment and Childhood): Gipuzkoa, Menorca, Sabadell and Valencia. The overall aim of the INMA project is to study the effect of environmental pollutants during early life (pregnancy and childhood) on growth, development and children’s health. From 1997 to 2008, a total of 2750 women were randomly recruited at the public hospital of reference in each region during the standard antenatal care visit in the first trimester of pregnancy [28]. The inclusion criteria were: ≥16 years old, intention to deliver in the reference hospital, singleton pregnancy, and no assisted conception or communication issues. Children and their families were followed during pregnancy (28–32 weeks of gestation), at delivery, and at the child’s age of 6 months, 1, 2, 4, 7, 9 and 11 years [28]. Of the initial 2750 women, a total of 2027 (74% of the initial cohort) had maternal vitamin D levels measured during pregnancy and available data of at least one body composition or growth outcome at 7 or 11 years. Therefore, the final sample size varied depending on the outcome: BMI at 7 years or 11 years (*n* = 1594), fat mass (*n* = 1375) at 11 years, or BMI trajectories from birth until 11 years (*n* = 1738) (Figure S1). The study was approved by the regional ethical committees of each cohort, and all participants signed written informed consent [28].

### 2.2. Vitamin D

Quantification of vitamin D metabolic status was assessed by determining circulating plasma levels of 25-hydroxyvitamin D_3_ (25(OH)D3, referred to as vitamin D_3_ in this manuscript). A single blood specimen was drawn during pregnancy (mean = 12.9 weeks of gestation [standard deviation (SD = 2.3)]). Samples were immediately processed after collection and stored at −80 °C. Quantification of maternal 25(OH)D3 levels was performed by high-performance liquid chromatography (HP-LC) using a BioRAD kit according to “Clinical and Laboratory Standard Institute” protocols in Muchen (Germany) [29]. The detection limit was 5 ng/mL and the variation coefficient between assays was 4.5%. The assay was validated by German Programmes of External Evaluation of Quality, and results were satisfactory in 100% of the cases. To remove the effect of the season when the blood was drawn was obtained, we used deseasonalized vitamin D concentrations [30] (see online supplement material Methods I and Figure S2).

### 2.3. Outcome Assessment

Anthropometric measurements. Height and weight were measured using standard protocols by trained staff. We calculated BMI (weight in kilograms/length in square meters) and age- and sex-standardized z-scores based on World Health Organization reference values [31,32]. Overweight was defined as the BMI z-score equal to or above the 85th percentile [32]. Information was available for the four regions included (Gipuzkoa, Sabadell, Valencia and Menorca) at 7 and 11 years.

Body composition. Tetrapolar bioelectric impedance analyses were conducted using RJL device at 11 years in all regions. Fat-free mass was determined by using the Horlick equation [33]. Body fat was obtained by subtracting fat-free mass from measured total body weight. Body fat% was calculated as body fat (kg) divided by the total body weight (kg) and multiplied by 100.

BMI trajectories. We extended the previously created zBMI trajectories from birth to 9 years until 11 years (mean = 11.1 years, SD = 0.5) [34]. BMI data was extracted from medical records and measurements taken by trained INMA staff using standardized protocols. The average number of measurement points per child was 12.7. Five BMI z-score trajectories were estimated using latent class growth analysis (LCGA) [27,34]. (Figure S3); class 1, characterized by higher birth size followed by accelerated BMI gain; class 2, higher birth size, slower BMI gain; class 3, lower birth size, accelerated BMI gain; class 4, average size at birth, slower BMI gain (reference category in our analyses); and class 5, lower birth size, average BMI gain. Menorca was not included in this analysis because information on zBMI trajectories was not available (Table S1).

### 2.4. BMI Polygenic Risk Scores

We calculated BMI polygenic risk scores (PRSs) for 1523 European ancestry offspring. Of these, 1318 also had vitamin D levels measured during pregnancy and were included in the analysis. A total of 10 PRSs were calculated using the reference GWAS of BMI retrieved from the PanUK Biobank—European population—phencode 23104 (https://pan.ukbb.broadinstitute.org/ accessed on 16 May 2022) and the PRSice v2 tool [35] as described in online Supplementary Material II. Then, we ran linear regression models between z-score BMI at 7 years and each of the PRSs adjusting for the 10 first GWAS principal components (PCs). The PRS with the highest model-fit (R2 = 6.6%), which included 4474 single-nucleotide polymorphisms (SNPs), was considered the best and used in downstream analyses. This PRS was centered and scaled by subtracting the mean and dividing by the standard deviation and then was categorized into three groups: PRS-low (<25% percentile), PRS-mid (from 25 to 75% percentile), and PRS-high (>75% percentile). The PRS-high represented the group of children with the highest genetic predisposition to have high BMI (Figure S4).

### 2.5. Covariates

Maternal and child characteristics were obtained through questionnaires administered to the mothers during pregnancy (maternal ethnicity, country of birth, age at delivery, pre-pregnancy BMI, maternal education, maternal social class (coded according to the International Standard Classification for Occupations-88 system), active smoking during pregnancy, physical activity, adherence to the Mediterranean diet (rMED) and parity) or at 1 year of the child (duration of breastfeeding (any)). The children’s sex, birth weight and gestational age were obtained from medical records.

### 2.6. Statistical Analysis

We tested the shape of the relationship between deseasonalized pregnancy vitamin D_3_ levels and the different outcomes by General Additive Models (GAMs). None of the associations departed from linearity (Figure S5). In subsequent analyses, 25(OH)D3 levels were treated both as a continuous variable (per 5 ng/mL decrease [36,37]) and as a categorical variable: deficient vitamin D_3_ levels < 20 ng/mL, adequate vitamin D_3_ levels ≥ 20 ng/mL. In order to avoid the loss of participants in the study and associated bias, we performed multiple imputations of missing values in covariate data (<5%) (Table S2) that generated twenty imputed datasets. Variable transformations were applied to reach normality (otherwise variables were categorized) before imputing missing data using chained equations. Representative groups of outcomes were considered as predictors [38]. In the analyses described below, results from each imputed dataset were combined using Rubin’s rules.

Multivariable linear regression models were used to estimate β-coefficients and 95% confidence intervals (CIs) for the association between vitamin D_3_ levels and continuous outcomes in the offspring (z-score BMI at 7 and 11 years and percentage of body fat at 11 years). Multivariate logistic regression models were used to estimate the associations of binary outcomes (overweight at 7 and 11 years); odds ratios (OR) and 95% CIs were reported. Finally, multinomial logistic regression models were used to estimate associations with z-score BMI trajectories; relative risk rations (RRR) and 95% CIs were reported. We used Direct Acyclic Graphs (DAG) to identify the minimum set of confounders to adjust the variables (Figure S6). Models were adjusted for region of residence, maternal country of birth, maternal age at delivery, maternal pre-pregnancy BMI, maternal education, maternal social class, maternal smoking during pregnancy, and parity.

Sensitivity analyses were performed to assess the robustness of the results. All models were repeated using the complete case dataset. In addition, we ran all models adjusting for physical activity and adherence to the Mediterranean diet during pregnancy to analyze whether they potentially confounded the associations; these analyses excluded Menorca because information on these two factors was not collected during pregnancy. We tested possible effect modification by sex, pre-pregnancy BMI (under-normal weight vs. overweight-obese), smoking during pregnancy (no vs. yes smoking during pregnancy) and child polygenic risk score for increased susceptibility to higher BMI (low, medium, high) by including an interaction term in the models and performing stratified analysis by categories of these variables.

Analyses were conducted using R version 4.0.2 (R Foundation, Vienna, Austria). Statistical significance was set at *p*-value < 0.05 for multivariate analyses and *p*-value < 0.1 for the interaction test.

## 3. Results

### 3.1. Characteristics of the Population

Maternal and child characteristics for the study population are shown in Table 1. Mothers were, on average, 30 years old at the time of delivery and were predominantly of Spanish origin (92%). Twenty-five percent of mothers were overweight or obese before pregnancy and more than 30% smoked at the beginning of pregnancy. Median maternal vitamin D levels were 30.0 ng/mL (range: 22.7–37.3 ng/mL) and a total of 17.5% of the mothers had deficient vitamin D_3_ levels (<20 ng/mL) (Table S3). Mothers who had deficient levels during pregnancy were more likely to be born outside Spain, were younger, lower educated and smoked more during pregnancy compared to mothers with adequate levels. On the other hand, they were more likely to be from a higher social class (Table 1). Differences between mothers included and excluded can be found in Table S3.

The prevalence of being overweight was higher in boys (~42%) than in girls (~37%) at both ages. On the contrary, the average fat mass at 11 years was higher among girls (~27%) than boys (~24%) (Table 2). Regarding BMI z-score growth trajectories, most of the children (~32%) belonged to class 4 (reference trajectory), and the percentages of boys and girls classified in the different trajectories were quite similar. (Table 2). Finally, greater genetic susceptibility to a high BMI was associated with a higher BMI, percentage of fat mass and prevalence of overweight (e.g., overweight at 11 years was 28.6% in the low category, 38.4% in the medium and 56.4% in the high) (Table S4).

### 3.2. Vitamin D Associations with Body Composition Outcome and zBMI Trajectories

Continuous vitamin D_3_ levels during pregnancy were not associated with any of the outcomes in the overall study population (Table 3). However, being exposed to deficient vitamin D_3_ levels (<20 ng/mL) during pregnancy was associated with higher BMI z-score at 7 years (β = 0.16; 95% CI: 0.01, 0.32) and, with borderline statistical significance, at age 11 years (β = 0.15; 95% CI: −0.01, 0.32). Children of mothers with vitamin D deficiency during pregnancy also showed higher odds of being overweight, but these associations did not reach statistical significance (7 years: OR = 1.25;95% CI: 0.93, 1.68; 11 years: OR = 1.30; 95% CI: 0.96, 1.76). All associations between pregnancy vitamin D_3_ levels and childhood body composition measures showed evidence for interactions with sex (*p* for interactions < 0.1), which is why sex-stratified results are shown in Table 3. Associations were all consistently stronger in boys than in girls, and most reached statistical significance in boys, whereas they were close to the null in girls (Table 3). For example, in boys, deficient vitamin D status was associated with increments in BMI z-score, fat mass, and the odds of being overweight at 7 and 11 years (e.g., overweight OR 7 years = 1.76; 95% CI: 1.18, 2.64 and overweight OR 11 years = 1.68; 95% CI: 1.10, 2.56).

No statistically significant associations between pregnancy continuous vitamin D_3_ levels and zBMI trajectories were observed, either in the overall population or in the sex-stratified analyses (Table 4). In boys, being exposed to deficient prenatal vitamin D levels was associated with an increased risk of belonging to trajectory 3, which is characterized by lower birth size and accelerated BMI gain (Relative Risk Ratio (RRR) = 1.76; 95% CI: 1.02–3.02), whereas in girls deficient vitamin D status tended to be associated with a decreased risk of belonging to this trajectory 3 (RRR = 0.62; 95% CI: 0.32–1.20) (*p* for interactions 0.1).

### 3.3. Sensitivity and Effect Modification Analyses

Analyses using complete-case data sets (Tables S5 and S6) and adding the covariates “adherence to Mediterranean diet” and “physical activity” yielded similar results (Table S7).

Regarding effect modification analyses, there was generally little evidence of interaction by maternal smoking during pregnancy or by maternal pre-pregnancy weight status (*p* for interactions > 0.1, Table S8). It was only in mothers with normal weight that deficient vitamin D levels in pregnancy were associated with an increase in body fat percentage in their children at 11 years (β = 1.23; 95% CI: −0.03, 2.49), while in mothers with overweight the association was in the opposite direction (β = −1.04; 95% CI: −3.35, 1.27) (*p* for interactions 0.08).

**Figure 1:**
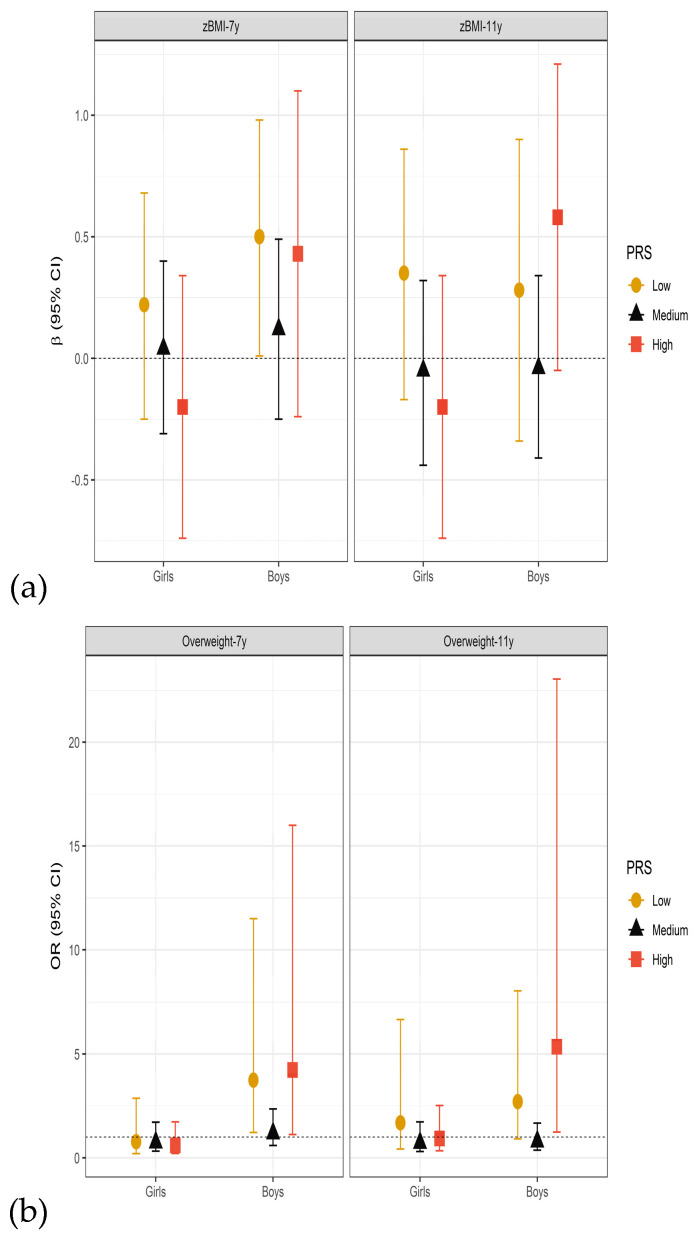
Plot of the associations between maternal vitamin D_3_ levels (deficient vs. adequate) and zBMI at 7 and 11 years (**a**) and overweight at 7 and 11 years (**b**) divided by sex and categorized by the polygenic risk score categories (low, medium and high). The dotted line at the value of 1 on the *y*-axis typically represents the reference line. The value of 1 is the neutral point for an odds ratio. The reference line at 1 on the *y*-axis helps visually compare the effects of different categories or groups relative to the reference group in terms of odds ratio.

We also observed little evidence for interactions with the PRS we created for increased genetic susceptibility to a higher BMI in the overall study population (*p*-values for interaction generally >0.1) (Table S9). However, when we further explored the PRS results separately for boys and girls (Figure 1 and Table S9), we observed that the associations between vitamin D deficiency and percentage of fat mass and overweight at 11 years were stronger in boys with high genetic predisposition (fat mass percentage β = 6.89; 95% CI: 2.69, 11.10, overweight OR = 4.23; 95% CI: 1.12, 16.00, *p* interactions, respectively, 0.01 and 0.07).

## 4. Discussion

In this prospective population-based birth cohort study in Spain, we found that maternal vitamin D levels during pregnancy were associated with child body composition, but only in boys. Specifically, we observed that deficient (<20 ng/mL) vitamin D_3_ levels were associated with higher BMI z-score, a higher percentage of fat mass and higher odds of being overweight compared to those boys exposed to adequate vitamin D_3_ levels. No consistent associations were found in girls. In addition, we found evidence that this association in boys might be influenced by the genetic predisposition to a higher BMI of the child. We found no significant associations between continuous vitamin D_3_ levels and zBMI trajectories, although in boys, vitamin D deficiency was associated with an increased risk of belonging to the trajectory characterized by lower birth size and accelerated BMI gain.

### 4.1. Association between Early Pregnancy Maternal Vitamin D Levels and Child Body Composition

In concurrence with our study, some previous longitudinal studies that measured vitamin D levels in early-mid pregnancy also reported an association between maternal vitamin D and offspring body composition profiles [19,21,22,23,26,39,40]. In particular, low maternal vitamin D levels in early–mid-gestation have been previously associated with higher odds of being overweight at 1 year [21], and with an increment of fat mass percentage at 5–6 years [19,22,23,39,40] and at 11 years [26] (but only in active smoker mothers). However, evidence from studies that measured vitamin D in late pregnancy or cord blood is less consistent. While most of them did not find any evidence of an association [17,18,20,39], low vitamin D levels in late pregnancy were associated with greater fat mass at 4 and 6 years in one study [41]. A biologically plausible argument that may help to explain these findings can be related with to the timing of fetal adipose tissue development, which starts with the appearance of the first fat lobules at 14 weeks of gestation and is mainly finished by the 23rd week, when the total adipocyte number remains quite constant [42].

Recent studies in rodents support the role of maternal vitamin D deficiency during pregnancy in adipocyte and adipose tissue metabolic programming in the offspring [7,43,44]. Vitamin D deficiency in rats was associated with increased body weight, higher percentage of fat tissue and impaired glucose and lipid metabolism, and also caused epigenetic changes leading to this obese phenotype [44]. In a similar study in mice, vitamin D deficiency was associated with increased expression of adipogenesis-promoting genes such as PPARγ [43]. Indeed, the nuclear factor PPARγ is the convergence point of most adipogenic pathways and considered a prime regulator of adipogenesis. Nevertheless, previous evidence is not always consistent, and multiple differences exist on the effects of vitamin D deficiency on adiposity, metabolism or inflammation in the offspring [45].

### 4.2. Sex-Specific Associations

Compared with the literature, a surprising finding in our study was that the effects of vitamin D deficiency were only present in boys. In a previous study also conducted in the INMA cohort [21], deficient vitamin D levels were associated with an increased risk of being overweight in the offspring both in utero and in the first year of life, but the association was attenuated at 4 years. However, whether or not the results differed by sex was not tested. In fact, from the previous longitudinal studies, only a few evaluated whether or not results differed by sex with inconsistent results [19,22,39]. In agreement with the sex-differential findings from the present study, in a prospective cohort among 500 Indian mother–child pairs, lower maternal vitamin D concentrations in mid-gestation were associated with lower lean mass in the offspring and with higher fat percentage at 5 years but only in boys. They ascribed the differences to the distinct lifestyle habits of Indian boys and girls at this age. In the large Generation R cohort (N = 4900) in the Netherlands, severely deficient mid-gestation vitamin D levels were found to be associated with higher fat mass percentage and lower lean mass percentage at 6 years with no evidence of sex interaction [39]. Finally, in the RHEA cohort in Greece, low vitamin D levels in early pregnancy were associated with a higher body mass index and waist circumference in the offspring at 4 and 6 years and associations were found to be stronger among girls, but the sample size was small (N = 370) [22].

Even we do not have a clear explanation for the abovementioned discrepancies regarding the sex differences of maternal vitamin D effect on body composition in the offspring, there is evidence from in vitro and animal studies that support a sex-specific metabolic response to maternal vitamin D deficiency during pregnancy [46,47]. One explanation is that this relies on testosterone regulation of vitamin D metabolism in the placenta [46]. It has been suggested that testosterone diminishes the synthesis and/or increases the catabolism of calcitriol (the active metabolite of vitamin D) by affecting the expression of key genes involved in its synthesis or catabolism [46]. Furthermore, another study observed that 17β-estradiol plasma levels were significantly increased in serum of offspring females exposed to vitamin D deficiency in utero, which was not observed in offspring males [47]. Given the well-established link between estrogens and fat accumulation, the authors suggested that this finding may be one of the reasons for observing these sex-specific effects of vitamin D deficiency.

Consistent with results for body composition, we observed some differences between boys and girls in the association between vitamin D levels and BMI trajectories. While deficient 25(OH)D3 levels during pregnancy were associated with the trajectory 3, characterized by lower birth size and accelerated growth during childhood in boys; in girls, a reverse association with this trajectory was observed. In a recent systematic review and meta-analysis of studies that assessed the effect of prenatal vitamin D status on offspring’s growth, adiposity and metabolic health, it was found that low prenatal levels were associated with lower birth weight and accelerated growth and adiposity in postnatal life, but differences between sexes were not evaluated [48]. The trajectory of lower birth size and accelerated growth was recently associated with increased systolic and diastolic blood pressure in our INMA cohort [34]. In addition, an accelerated BMI gain has been associated with an increased risk of coronary events later in life by others [4]. Thus, further studies are needed to confirm pregnancy vitamin D deficient levels as a factor that predisposes children to follow a growth trajectory characterized by lower birth size and accelerated BMI gain and the associated consequences.

### 4.3. Other Potential Effect Modifiers

Previous studies identified pre-pregnancy BMI [22] and active smoking during pregnancy [26] as factors that could modify the association between vitamin D levels during gestation and body composition in the offspring. In our analyses, we did not observe any effect of smoking during pregnancy while, in agreement with the results of the RHEA Greek cohort [22], we observed some effect modification by pre-pregnancy BMI. In normal-weight women, deficient prenatal vitamin D levels were associated with higher offspring fat mass, whereas in overweight/obese women estimates were in the opposite direction, but not statistically significant. Although pre-pregnancy BMI categorization differed between the two studies (they compared normal weight and overweight vs. obese), results showed the same trend, which can reflect differences in vitamin D metabolism depending on the fat storage or distribution in pregnant women [49].

Although we did not observe a clear modifying effect of child genetic susceptibility to a higher BMI in the associations between pregnancy vitamin D and BMI, there was some tendency towards stronger associations in children with lower susceptibility to a higher BMI. However, when we looked at sex differences in an explorative analysis, associations were close to null in girls in all strata of genetic predisposition, whereas in boys, the associations were stronger, and statistically significant, in those with both low and high genetic predisposition. These results are based on small numbers of observations, so should be interpreted with caution and replicated in future studies. To our knowledge, no previous studies have evaluated the influence of genetic predisposition to higher BMI on associations between vitamin D and body composition.

### 4.4. Strengths and Limitations

A main strength of our study is that we followed children until 11 years of age, giving a longer follow-up period than most other studies and allowing us to evaluate if associations observed in early childhood persist into later childhood. Further, our study had a large sample size compared with most of the other studies, and included the evaluation of potential effect modification by variables identified in other studies and by genetic predisposition using a polygenic risk score which explained almost 7% of the BMI variability. We included in our outcome assessment the body fat mass percentage measured by bio-impedance, which gives a more direct and reliable measure of adiposity than just measuring BMI. Lastly, the multiple follow-up points throughout infancy and childhood and the use of latent class growth analysis enabled us to assign BMI trajectory classes to our population, allowing us to integrate multiple aspects of child growth and, compared to outcomes assessed in a single time point, which permit more accurate identification of young children at higher risk of disease.

The study also has some limitations. First, relying on only one vitamin D assessment during pregnancy may not be representative of the entire period. However, the adipose tissue starts to develop at the end of the first trimester of pregnancy [42], which corresponds more or less to the mean week of gestation in which blood samples were obtained. Additionally, we used 25(OH)D3 levels, which did not reflect circulating levels of 25(OH)D2 and may contribute to exposure misclassification. However, 25(OH)D levels are considered the most reliable indicator of vitamin D status [50], and most of the 25(OH)D is in 25(OH)D3 form. In addition, although vitamin D levels were assessed using high-performance liquid chromatography (HPLC) and not mass spectrometry (LC-MS), which considered the gold standard technique to assess vitamin D levels, there is evidence of a good agreement between both techniques in terms of accuracy and precision [51,52]. Regarding BMI trajectories, we could not include data from Menorca in our analysis and, therefore, we only used three cohorts. The sample size in these analyses was too limited to be used in the evaluation of effect modifiers by stratifying groups, so we only ran stratified analyses for sex since the other outcomes showed clear sex-specific associations. Also, we cannot rule out the potential residual confounding on the associations due to factors that we have not been able to measure, although adjusting the models for diet and physical activity did not have an influence on our associations. For example, individuals might differ in their capacity to obtain 1,25(OH)2D, which is the biologically active form of vitamin D and highly regulated by calcium levels. Therefore, there will always be inter-individual variability that we cannot measure and may confound the associations. In addition, future studies attempting to replicate the study could assess whether other factors, such as childhood diet or physical activity, have any influence on this association. Finally, loss-to-follow-up in our cohort may have led to selection bias; however, our previous work shows that this is unlikely to bias associations between vitamin D and child health outcomes [53], and we did not correct for multiple comparisons, which might have led to false-positive findings (type I error). However, statistical correction for multiple comparisons assumes that the tested hypotheses are independent, which is not the case in these analyses since the outcomes are correlated. Furthermore, it increases the chances of false negative findings (type II error), which might have worse consequences in the context of public health research.

## 5. Conclusions

Our results support a programming effect of early pregnancy vitamin D_3_ on offspring body composition. Being exposed to deficient vitamin D_3_ levels prenatally was associated with increased body mass index, overweight and fat mass in late childhood in a sex-specific manner. Prenatal vitamin D levels may be an early-modifiable risk factor to consider in order to prevent childhood overweight.

## Figures and Tables

**Table 1:** Maternal and child characteristics of the total included population and depending on the vitamin D_3_ levels during pregnancy: deficient (<20 ng/mL) and adequate (>20 ng/mL).

	Study Population				
		Total	Deficient	Adequate	
	% Missing	*n* = 2027	*n* = 355	N = 1672	*p*-Value
Maternal characteristics					
Region of residence; %	0				
Gipuzkoa		26.7	26.2	26.8	<0.001
Sabadell		27.4	37.5	25.5	
Valencia		32.4	21.9	34.4	
Menorca		13.5	14.4	13.3	
Maternal country of birth; % Spain	0.3	92.2	88.7	92.9	0.015
Maternal age; years (SD)	0	30.3 (4.2)	29.9 (4.4)	30.4 (4.2)	0.043
Maternal pregnancy BMI; kg/m^2^ (SD)	0.3	23.4 (4.2)	23.5 (4.3)	23.4 (4.2)	0.762
Maternal pregnancy weight status; %					
Underweight		4.4	6.6	4.0	0.093
Normal		70.8	66.1	71.7	
Overweight		17.5	19.7	17.0	
Obese		7.3	7.5	7.3	
Maternal education; %	0.5				
Primary or less		29.4	34.8	28.3	0.036
Secondary		38.8	38.2	39.0	
High		31.8	27.0	32.7	
Maternal social class; %	3.4				
Low		21.2	17.1	22.0	0.020
Medium		32.0	29.0	32.5	
High		46.8	53.9	45.5	
Maternal smoking pregnancy; %	1.9				
Never		67.7	65.1	68.2	0.033
Only early in pregnancy		15.8	13.7	16.3	
During whole pregnancy		16.5	21.3	15.5	
Parity; %	2.3				
No previous pregnancies		54.8	56.8	54.4	0.461
One or more		45.2	43.2	45.6	
Physical activity	14.6				0.449
Sedentary; %		5.4	6.6	5.2	
Slightly active; %		24.5	26.9	24.0	
Moderately active; %		42.6	41.7	42.7	
Quite active/very active; %		27.5	24.7	28.1	
Adherence Mediterranean diet	14.2				0.509
Low; %		42.3	41.0	42.5	
Medium; %		28.6	31.5	28.1	
High; %		29.1	27.5	29.4	
Gestational diabetes mellitus; % yes	15.7	4.6	5.2	4.4	0.686
Child characteristics					
Sex; % male	0	50.8	55.0	50.0	0.116
Birth weight; grams (SD)	0.4	3248 (475)	3261 (461)	3245 (478)	0.578
Gestational age; weeks (SD)	0.1	39.6 (1.6)	39.5 (1.6)	39.6 (1.6)	0.495
Preterm (<37 weeks); %	0.1	4.2	4.1	4.2	1
Breastfeeding duration; weeks (SD)	2.1	23.1 (20.1)	23.0 (20.2)	23.2 (20.0)	0.886

Abbreviations: Vitamin D_3_—25-hydroxyvitamin D_3_. Values are percentages for categorical variables and means (standard deviation) for continuous variables. *p*-value (deficient vs. adequate) was estimated by using One-Way ANOVA, Wilcoxon, or Chi-square tests. Non-imputed data.

**Table 2:** Child outcomes characteristics for the total population and distributed by sex.

	Total Study Population	Girls	Boys
	N = 2027	N = 997	N = 1030
Child outcome characteristics			
Age—7 years (years)	7.3 (0.6)	7.3 (0.6)	7.3 (0.6)
zBMI—7 years	0.74 (1.2)	0.68 (1.1)	0.80 (1.3)
Overweight—7 years: %	38.9	37.2	40.5
Age—11 years (years)	11.1 (0.5)	11.1 (0.5)	11.1 (0.5)
zBMI—11 years	0.65 (1.2)	0.57 (1.1)	0.74 (1.3)
Overweight—11 years: %	40.6	37.4	43.9
Fat mass—11 years (%)	25.5 (8.2)	26.6 (7.9)	24.3 (8.4)
BMI trajectories from birth to 11 years			
Class 1: higher birth size/accelerated gain	11.3	9.7	12.8
Class 2: higher birth size—slower gain	24.1	24.3	23.8
Class 3: lower birth size—accelerated gain	17.5	16.6	18.3
Class 4: average birth size—slower gain (ref.)	31.9	34.5	29.4
Class 5: lower birth size—slower gain	15.2	14.8	15.6

Abbreviations: zBMI—z-score body mass index. Values are percentages for categorical variables and means (standard deviation) for continuous variables. *p*-value (girls vs. boys) was estimated by using One-Way ANOVA, Wilcoxon, or Chi-square tests. Non-imputed data.

**Table 3:** Associations of maternal vitamin D_3_ levels, per 5 ng/mL decrease and deficient vs. adequate, with z-score body mass index at 7 and 11 years, percentage of fat mass at 11 years and overweight at 7 and 11 years for the total study population and then separately for female and male.

	**Total Study Population**			**Girls**			**Boys**		
	**5 ng/mL Decrease**		**Deficient**	**5 ng/mL Decrease**		**Deficient**	**5 ng/mL Decrease**		**Deficient**
	**N**	**β (95% CI)**	**β (95% CI)**	**N**	**β (95% CI)**	**β (95% CI)**	**N**	**β (95% CI)**	**β (95% CI)**
BMI z-score									
7 years	1536	0.01 (−0.02–0.04)	0.16 (0.01–0.32)	769	−0.02 (−0.05–0.02)	0.06 (−0.15–0.27)	767	0.04 (0.00–0.09)	0.29 (0.03–0.55)
11 years	1390	0.01 (−0.02–0.04)	0.15 (−0.01–0.32)	716	−0.02 (−0.06–0.02)	0.01 (−0.21–0.23)	674	0.04 (−0.01–0.09)	0.28 (0.03–0.52)
Body composition 11 years									
Body fat (%)	1375	0.07 (−0.13–0.26)	0.81 (−0.28–1.91)	709	−0.13 (−0.39–0.12)	0.01 (−1.50–1.52)	666	0.33 (0.04–0.63)	1.78 (0.24–3.32)
	**5 ng/mL decrease**		**Deficient**		**5 ng/mL decrease**	**Deficient**		**5 ng/mL decrease**	**Deficient**
	**N**	**OR (95% CI)**	**OR (95% CI)**	**N**	**OR (95% CI)**	**OR (95% CI)**	**N**	**OR (95% CI)**	**OR (95% CI)**
Overweight									
7 years	1536	1.00 (0.95–1.05)	1.25 (0.93–1.68)	769	0.95 (0.88–1.02)	0.83 (0.53–1.30)	767	1.07 (0.99–1.15)	1.76 (1.18–2.64)
11 years	1390	1.02 (0.96–1.08)	1.30 (0.96–1.76)	716	0.97 (0.90–1.05)	0.97 (0.61–1.53)	674	1.08 (0.99–1.17)	1.68 (1.10–2.56)

Abbreviations: Vitamin D_3_—25-hydroxyvitamin D_3_; CI—confidence intervals; β—Beta; OR—odds ratio; BMI—body mass index. Multivariable linear regression models for BMI z-score at 7 and 11 years and body composition at 11 years; logistic regression models for overweight at 7 and 11 years. Models were adjusted for region of residence, country of birth, age at delivery, pre-pregnancy body mass index, education, social class, smoking, and parity. Ref. Vitamin D_3_ category: adequate (>20 ng/mL).

**Table 4:** Associations of maternal Vitamin D_3_ levels, per 5 ng/mL decrease and deficient vs. adequate, with z-score body mass index trajectories from 0 to 11 years, for the total study population and separately for girls and boys.

	Total Study Population			Girls			Boys		
	5 ng/mL Decrease		Deficient	5 ng/mL Decrease		Deficient	5 ng/mL Decrease		Deficient
	N	RRR (95% CI)	RRR (95% CI)	N	RRR (95% CI)	β (95% CI)	N	RRR (95% CI)	β (95% CI)
zBMI trajectories	1.738			843			895		
1: higher birth size & accelerated BMI gain	197	1.01 (0.94–1.09)	1.15 (0.73–1.82)	82	1.02 (0.91–1.15)	0.89 (0.42–1.86)	115	1.00 (0.90–1.11)	1.49 (0.81–2.73)
2: higher birth size & slower BMI gain	418	1.05 (0.99–1.12)	1.23 (0.87–1.75)	205	1.07 (0.98–1.17)	1.08 (0.66–1.77)	213	1.04 (0.95–1.13)	1.41 (0.85–2.37)
3: lower birth size & accelerated BMI gain	304	1.02 (0.95–1.09)	1.11 (0.74–1.66)	140	0.95 (0.87–1.05)	0.62 (0.32–1.20)	164	1.08 (0.98–1.19)	1.76 (1.02–3.02)
5: lower birth size & average BMI gain	265	0.99 (0.93–1.06)	0.83 (0.53–1.30)	125	1.00 (0.91–1.11)	0.70 (0.37–1.33)	140	0.98 (0.89–1.08)	1.01 (0.54–1.91)

Abbreviations: Vitamin D_3_—25-hydroxyvitamin D_3_; CI—confidence intervals; RRR—relative risk ratio; BMI—body mass index. Multinomial logistic regression models. Models were adjusted for region of residence, country of birth, age at delivery, pre-pregnancy body mass index, education, social class, smoking, and parity. Ref. Vitamin D_3_ category: adequate (>20 ng/mL), Ref. zBMI trajectory: 4 average birth size and slower BMI gain.

## Data Availability

Data described in the manuscript, code book, and analytic code will be made available upon request pending. INMA external access procedures are described: https://www.proyectoinma.org/en/inma-project/inma-collaboration-policy/ (accessed on 21 March 2023).

## Supplementary Materials

The following supporting information can be downloaded at: https://www.mdpi.com/article/10.3390/nu15092033/s1. Figure S1. Flowchart of the study population; Methods I/Figure S2. Deseasonalization of 25(OH)D3 levels Figure S3. BMI trajectories from 0 to 11 years; Methods II/Figure S4. Genome-Wide Genotyping, Quality Control, and Imputation and polygenic risk score calculation; Figure S5. Smooth association between maternal 25(OH)D3 and growth and body composition: z-score body mass index at 7 years (1) and 11 years (2), percentage of fat mass (3) and total fat mass (Kg) (4) at 11 years and overweight at 7 years (5) and 11 years (6); Figure S6. Directed Acyclic graph of the association of maternal vitamin D and growth and body composition outcomes. Table S1. Description of the growth and body composition child’s characteristics according to region of residence; Table S2. Missing data of the variables imputed by region of residence; Table S3. Maternal and child characteristics of the included and excluded populations for the associations of maternal 25(OH)D3 during pregnancy and growth and body composition outcomes; Table S4. Description of the growth and body composition child’s characteristics according to the polygenic risk score category; Table S5. Complete-case analysis (minimally and fully adjusted) of maternal 25(OH)D3 levels, per 5 ng/mL decrease and deficient vs. adequate, with z-score body mass index at 7 and 11 years, percentage of fat mass at 11 years and overweight at 7 and 11 years; Table S6.Complete-case analysis (minimally and fully adjusted) of maternal 25(OH)D3 levels, per 5 ng/mL decrease and deficient vs. adequate, with z-score body mass index trajectories from 0 to 11 years; Table S7. Association of maternal 25(OH)D3 levels, per 5 ng/mL decrease and deficient vs. adequate, with z-score body mass index at 7 and 11 years, percentage of fat mass at 11 years and overweight at 7 and 11 years; Table S8. Associations of maternal 25(OH)D3 levels, per 5 ng/mL decrease and deficient vs. adequate, with z-score body mass index at 7 and 11 years, percentage of fat mass at 11 years and overweight at 7 and 11 years for pre-pregnancy body mass index categories (normalwight and overweight) and smoking during pregnancy (no and yes); Table S9. Associations of maternal 25(OH)D3 levels, per 5 ng/mL decrease and deficient vs. adequate, with z-score body mass index at 7 and 11 years, percentage of fat mass at 11 years and overweight at 7 and 11 years for the different categories of the created polygenic risk score for higher BMI and separately by sex of the child. References [54,55,56,57,58,59,60,61,62] are cited in Supplementary Materials.
