# Supplementary material for: Prenatal Vitamin D Levels Influence Growth and Body Composition until 11 Years in Boys
Source: Nutrients. 2023 Apr 23;15(9):2033. doi: 10.3390/nu15092033 (PMC10181475; doi:10.3390/nu15092033)
Supplement: Supplementary file 1 [file nutrients-15-02033-s001.zip › nutrients-2304697-SUPPLEMENTARY MATERIAL SI_vitaminD_GBD.pdf]

SUPPLEMENTARY MATERIAL SI

Content:

Figure S1. Flowchart of the study population.

Methods SI / Figure S2. Deseasonalization of 25(OH)D3 levels

Figure S3. BMI trajectories from 0 to 11 years

Methods SII / Figure S4. Genome-Wide Genotyping, Quality Control, and Imputation and polygenic risk score calculation.

Figure S5. Smooth association between maternal 25(OH)D3 and growth and body composition: z-score body mass index at 7 years (1) and 11 years (2), percentage of fat mass (3) and total fat mass (Kg) (4) at 11 years and overweight at 7 years (5) and 11 years (6).

Figure S6. Directed Acyclic graph of the association of maternal vitamin D and growth and body composition outcomes.

References

Figure S1. Flowchart of the study population

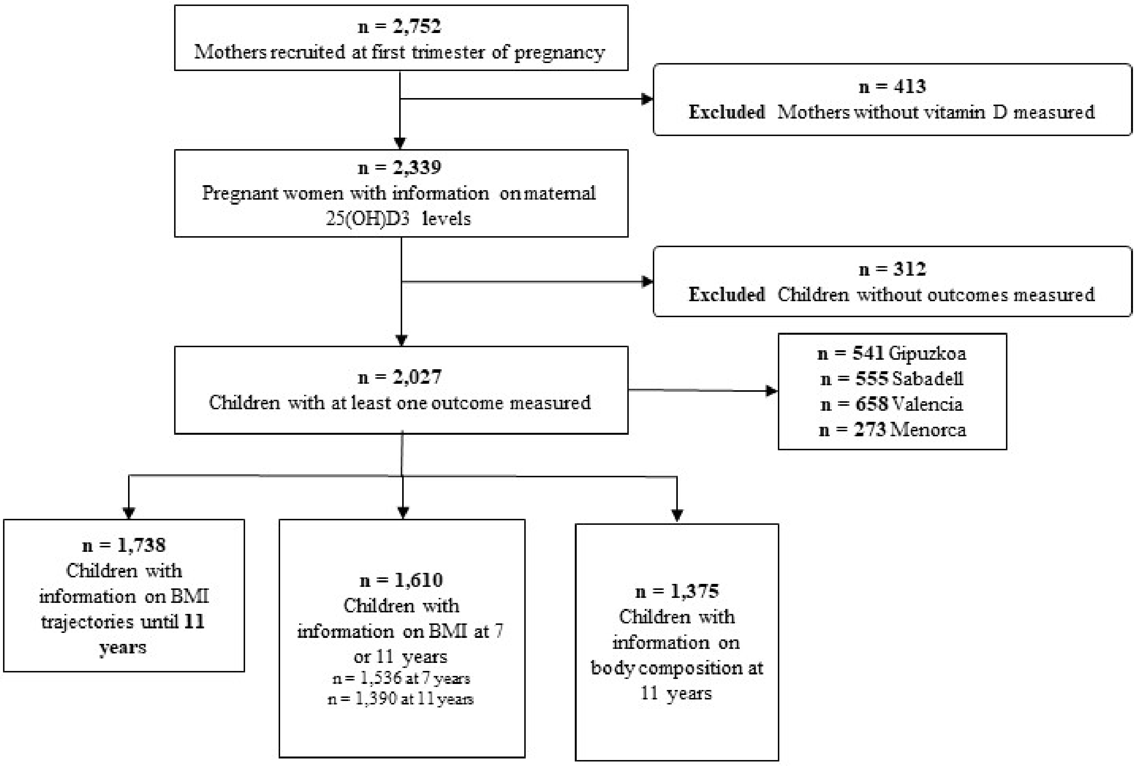

METHODS SI

<u>Deseasonalization of 25(OH)D_3 levels</u>

Seasonality of 25(OH)D_3 was tested by fitting the data to a sine function with a period of 12 months in a nonlinear regression cosinor model (1). Then, the predicted 25(OH)D_3 concentrations based on the months at blood collection for each subject, derived from the sinusoidal model, were substracted from the actual observed value (Figure S1). Subsequently, the overall mean was added and the resulting deseasonalized 25(OH)D_3 concentrations were analyzed.

Figure S2. Fitted sinusoidal model for observed maternal circulating 25(OH)D_3 levels.

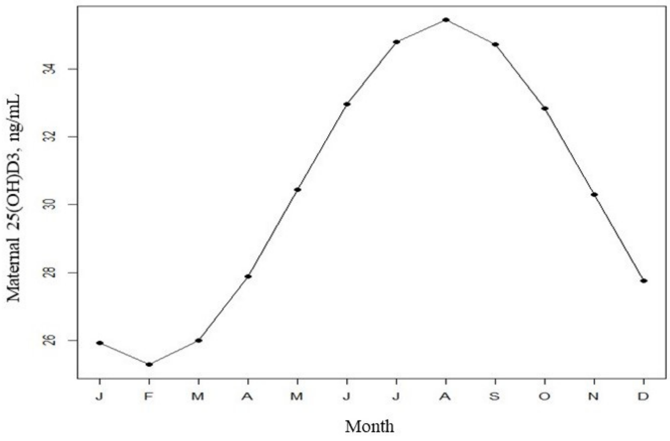

Figure S3. BMI trajectories from 0 to 11 years

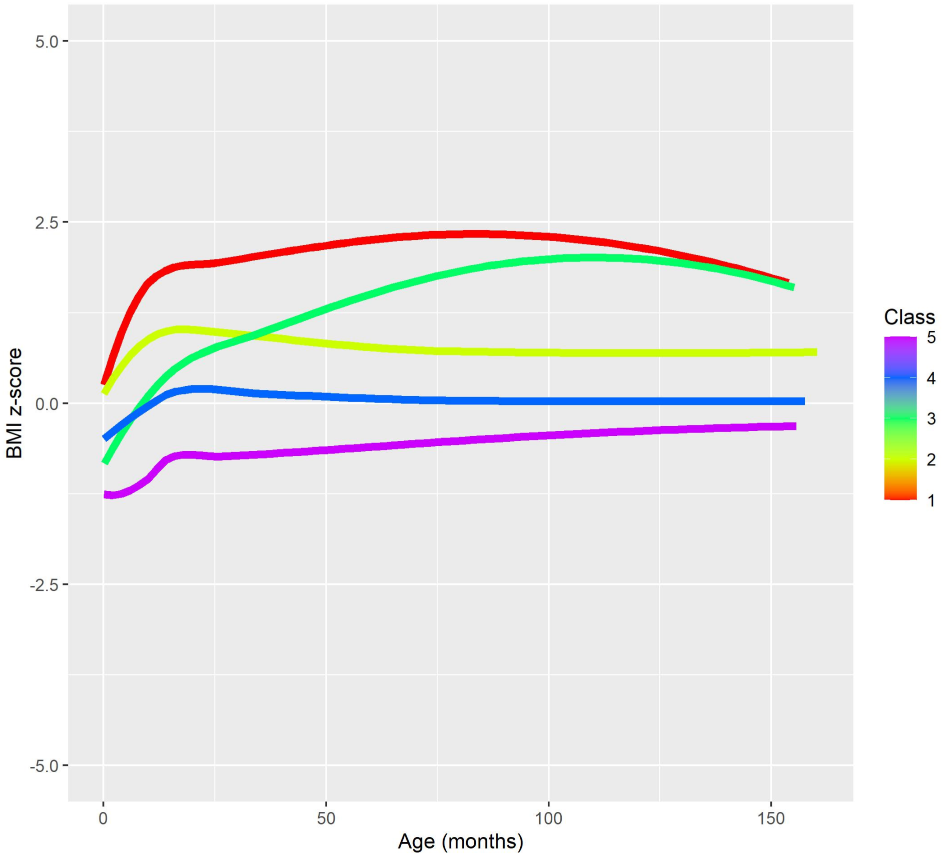

Description of the BMI z-score trajectory classes: class 1, higher birth size and accelerated gain; class 2, higher birth size and slower gain; class 3, lower birth size and accelerated gain; class 4, average birth size and slower gain (reference), class 5, lower birth size and slower gain.

METHODS SII

Genome-wide genotyping, quality control, and imputation

DNA was obtained from placenta (fetal side), cord blood at birth, whole blood at age 4y or 9y, or saliva at age 4y at the Spanish National Genotyping Center (CEGEN-Barcelona) using the Chemagen kit (PerkinElmer) or the Basque Biobank (Biobanko-Donostia/San Sebastian) using the Flexigene DNA kit (Qiagen). DNA samples were quantified with the the Quant-iT PicoGreen dsDNA Assay Kit (Life Technologies) and their quality was evaluated in a subset of them using a NanoDrop Spectrophotometer (Thermo Scientific) and by random visualization on agarose gels. Working plates at 50 ng/ul were prepared with available automation workstations.

Children were genotyped using two arrays. On one hand, 1,081 children from INMA Sabadell, Menorca and Valencia and whose parents reported to be of European ancestry and to be born in Spain or in European countries and that were not lost during the 4y follow-up were selected for genome-wide genotyping with the HumanOmni1-Quad v1.0 Beadchip (Illumina) at the Spanish National Genotyping Center (CEGEN-Barcelona). On the other hand, a total of 1,350 children from INMA Sabadell, Gipuzkoa, Granada and Valencia were genome-wide genotyped using different versions of the Illumina GSA Beadchip at the Human Genotyping Facility (HuGeF), Dept Internal Medicine, Erasmus MC, Rotterdam, The Netherlands and the Spanish National Genotyping Center, CEGEN, Madrid, Spain.

Quality control (QC) in each dataset was performed with the PLINK program following standard recommendations (2–4). Variants with a call rate below 95%, minor allele frequency below 1%, or a P-value from a Hardy-Weinberg exact test below 1e-06 were removed. Samples with discordant sex, those with average heterozygosity values above or below 3 (Omni1) or 4 (GSA) standard deviations, or with more than 2% (Omni1) or 3% (GSA) missing genotypes were filtered out. Identity-by-descent values were calculated with PLINK, and from those sample pairs related at the approximate level of first cousins (PI-HAT estimates above 0.18) the sample with higher proportion of missing genotype was removed.

Genotype imputation was performed with the Michigan Imputation Server (5) using the Haplotype Reference Consortium reference panel (Version r1.1 2016) (6) in each dataset separately (GSA vs Omni1). Before imputation, data was converted into VCF format. Phasing of haplotypes was done with Eagle v2.4 (7) and genotype imputation with Minimac4 (8), both implemented in the code by the Michigan Imputation Server. In total, we retrieved ~40M variants after imputation, with around 1% of them being genotyped. In each imputed dataset, markers with imputation quality $R^2 > 0.9$ and MAF > 0.01 were kept. The intersection between both datasets was performed with bcftools (9) creating a final VCF file with 3,668,324 genetic variants and 1,809 samples (for INMA Sabadell we kept data obtained with the GSA array). Then, this VCF files was converted to plink 1.9 binary format. Principal Components from the GWAS data were calculated for the full dataset and ancestry was estimated using the peddy program (10).

Polygenic Risk Score Calculation and Validation

PRSs for BMI were computed for 1,523 children of the European ancestry using PRSice v2 program (11) and the imputed quality controlled genetic dataset. Summarized results of reference GWAS (base data) were retrieved from the PanUK Biobank – European population - phencode 23104 (https://pan.ukbb.broadinstitute.org/). PRSs were calculated operating with the average score method, which computes the score as a sum of the summary statistics for the effective allele multiplied by the number of effective alleles observed, divided by the numbers of alleles included in the PRS. SNP clumping was set at $r2 > 0.1$, and the rest of the arguments were left as default in PRSice v2. We calculated 10 PRSs based on ten different p-value thresholds (Pt) of the base GWAS for SNP inclusion: Pt = {0.00000005, 0.000005, 0.0001, 0.001, 0.01, 0.05, 0.1, 0.2, 0.5, and 1}. The overlap of genetic variants between the base GWAS and the target GWAS was 3 M, and 91,751 were kept after clumping for linkage disequilibrium.

Figure S4. Boxplot of the association between the 3 categories of the PRS and the z-score body mass index at 7 years.

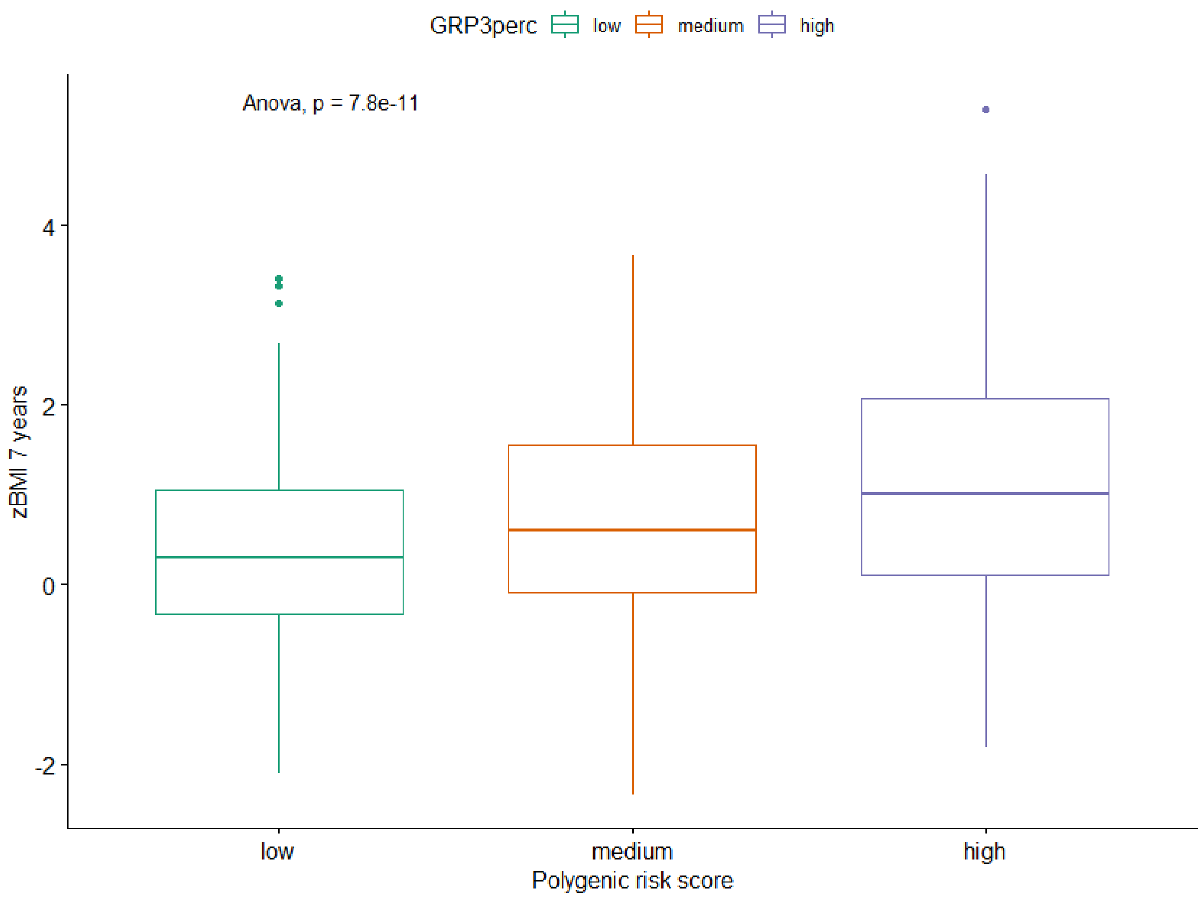

Figure S5. Smooth association between maternal 25(OH)D3 and growth and body composition: z-score body mass index at 7 years (1) and 11 years (2), percentage of fat mass (3) and total fat mass (Kg) (4) at 11 years and overweight at 7 years (5) and 11 years (6).

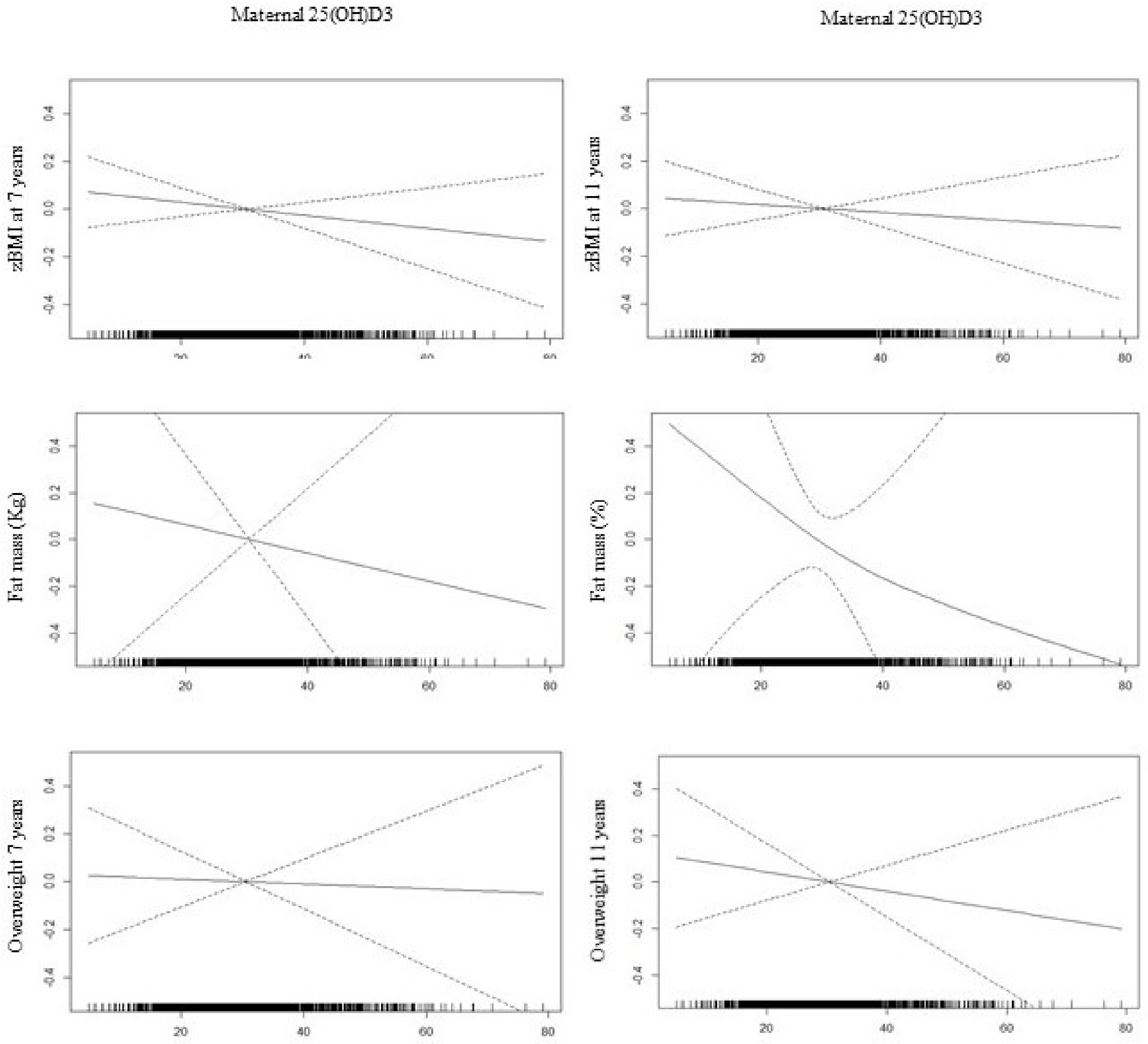

Figure S6. Directed Acyclic graph of the association of maternal vitamin D and growth and body composition outcomes.

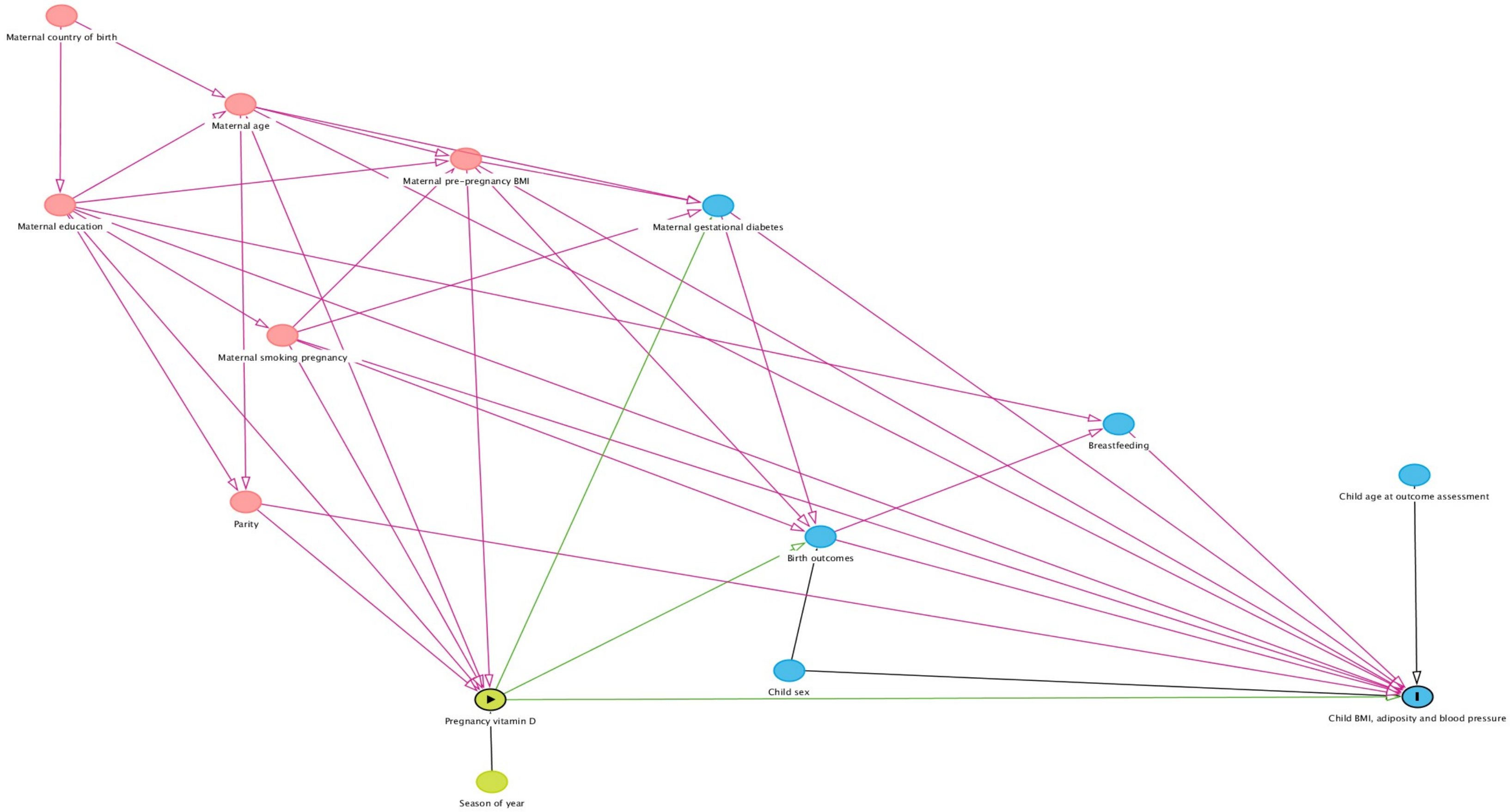

White nodes are variables associated with both the exposure and the outcome for which the models have been adjusted; blue nodes are variables associated with the outcome; green nodes are variables associated with the exposure.
Maternal socioeconomic status includes maternal country of birth, maternal education, and maternal social class.
DAGitty version 3.0 (12)

Bibliography

1. Morales E, Guxens M, Llop S, et al. Circulating 25-hydroxyvitamin D3 in pregnancy and infant neuropsychological development. Pediatrics 2012;130:e913-2.

2. Chang CC, Chow CC, Tellier LCAM, Vattikuti S, Purcell SM, Lee JJ. Second-generation PLINK: Rising to the challenge of larger and richer datasets. Gigascience 2015;4:1–16.

3. Purcell S, Neale B, Todd-Brown K, et al. PLINK: A tool set for whole-genome association and population-based linkage analyses. Am J Hum Genet 2007;81:559–75.

4. Anderson CA, Pettersson FH, Clarke GM, Cardon LR, Morris AP, Zondervan KT. Data quality control in genetic case-control association studies. Nat Protoc 2010;5:1564–73.

5. Das S, Forer L, Schönherr S et al. Next-generation genotype imputation service and methods. Nat Genet 2016;48:1284-1287.

6. McCarthy S, Das S, Kretzschmar W, et al. A reference panel of 64 , 976 haplotypes for genotype imputation. Nat Genet 2016;48:1279–83.

7. Loh P, Danecek P, Palamara PF, et al. Reference-based phasing using the Haplotype Reference Consortium panel. Nat Genet 2016;48:1443–8.

8. Fuchsberger C, Abecasis GR, Hinds DA. Minimac2: Faster genotype imputation. Bioinformatics 2015;31:782–4.

9. Verma SS, de Andrade M, Tromp G, et al. Imputation and quality control steps for combining multiple genome-wide datasets. Front Genet 2014;5:1–15.

10. Pedersen BS, Quinlan AR. Who's Who? Detecting and Resolving Sample Anomalies in Human DNA Sequencing Studies with Peddy. Am J Hum Genet 2017;100:406–13.

11. Choi SW, O'Reilly PF. PRSice-2: Polygenic Risk Score software for biobank-scale data.

Gigascience 2019;8:1–6.
